# Doxorubicin-Loaded PLGA Nanoparticles for Cancer Therapy: Molecular Weight Effect of PLGA in Doxorubicin Release for Controlling Immunogenic Cell Death

**DOI:** 10.3390/pharmaceutics12121165

**Published:** 2020-11-29

**Authors:** Yongwhan Choi, Hong Yeol Yoon, Jeongrae Kim, Suah Yang, Jaewan Lee, Ji Woong Choi, Yujeong Moon, Jinseong Kim, Seungho Lim, Man Kyu Shim, Sangmin Jeon, Ick Chan Kwon, Kwangmeyung Kim

**Affiliations:** 1 KU-KIST Graduate School of Converging Science and Technology, Korea University, 145 Anam-ro, Seongbuk-gu, Seoul 02841, Korea; 113377@kist.re.kr (Y.C.); 218312@kist.re.kr (J.K.); haehwan@kist.re.kr (S.Y.); 220343@kist.re.kr (J.L.); 217802@kist.re.kr (J.W.C.); 218843@kist.re.kr (J.K.); 2 Center for Theragnosis, Biomedical Research Institute, Korea Institute of Science and Technology (KIST), Seoul 02792, Korea; seerou@kist.re.kr (H.Y.Y.); phoenix0310@kist.re.kr (Y.M.); lim@kist.re.kr (S.L.); mankyushim@noxpharm.co.kr (M.K.S.); jeon@kist.re.kr (S.J.)

**Keywords:** cancer immunotherapy, immunogenic cell death, nanomedicine, drug release

## Abstract

Direct local delivery of immunogenic cell death (ICD) inducers to a tumor site is an attractive approach for leading ICD effectively, due to enabling the concentrated delivery of ICD inducers to the tumor site. Herein, we prepared doxorubicin (DOX)-loaded poly(lactic-co-glycolic acid) (PLGA) nanoparticles (NPs) using different molecular weight PLGA (7000 g/mol and 12,000 g/mol), showing different drug release kinetics. The different release kinetics of DOX might differently stimulate a tumor cell-specific immune response by releasing damage-associated molecular patterns (DAMPs), resulting in showing a different antitumor response in the living body. DOX-PLGA_7K_ NPs showed faster DOX release kinetics than DOX-PLGA_12K_ NPs in the physiological condition. DOX-PLGA_7K_ NPs and DOX-PLGA_12K_ NPs were successfully taken up by the CT-26 tumor cells, subsequently showing different DOX localization times at the nucleus. Released DOX successfully lead to cytotoxicity and HMGB1 release in vitro. Although the DOX-PLGA_7K_ NPs and DOX-PLGA_12K_ NPs showed different sustained DOX release kinetics in vitro, tumor growth of the CT-26 tumor was similarly inhibited for 28 days post-direct tumor injection. Furthermore, the immunological memory effect was successfully established by the ICD-based tumor-specific immune responses, including DC maturation and tumor infiltration of cytotoxic T lymphocytes (CTLs). We expect that the controlled release of ICD-inducible chemotherapeutic agents, using different types of nanomedicines, can provide potential in precision cancer immunotherapy by controlling the tumor-specific immune responses, thus improving the therapeutic efficacy.

## 1. Introduction

Cancer immunotherapy strategies, which can inhibit tumor growth and prevent its recurrence with metastasis, have been widely developed for the effective treatment of tumors by stimulating the immune systems in the body [1,2]. Various cancer immunotherapy strategies, including cytokine therapy, adoptive T-cell therapy, immune checkpoint blockade therapy, and cancer vaccines, have shown attractive results in clinics [3,4]. Conventional cancer vaccines, such as peptides and protein antigens, are widely used to stimulate immune responses against the tumors. However, they showed diverse efficacies between different types of tumors due to heterogeneous antigen expression of the tumor cells. Furthermore, therapeutic responses of cancer vaccines are still insufficient due to the immunosuppressive tumor microenvironment, including neovascularization, increments in immunosuppressive cells, and expression of inhibitory molecules [5].

Recently, specific chemotherapeutic agents, such as doxorubicin (DOX), oxaliplatin, mitoxantrone, and cyclophosphamide, showed that they could induce immunogenic cell death (ICD), resulting in eliciting antitumor immunity against tumor antigens [6]. The ICD-related multiple cell death pathways of tumor cells lead to exhibit damage-associated molecular patterns (DAMPs), including calreticulin (CRT) translocation, adenosine triphosphate (ATP) secretion, and high mobility group box 1 (HMGB1) release [7,8]. The characteristic molecules in the DAMPs stimulate antitumor immunity by maturating dendritic cells (DCs) and activating antigen-specific cytotoxic T lymphocytes (CTLs), resulting in killing the tumor cells. Based on the vaccine-like role of ICD, several chemotherapeutic agent-encapsulated nanoparticles have been used as in situ cancer vaccines, resulting in successfully eliciting antitumor immune responses against various types of heterogeneous tumors [9].

Nanoparticles (NPs) based on PLGA are biocompatible and biodegradable, and can sustain the release of various antigens as well as anticancer agents for effective treatment of tumors [1,10,11]. In particular, the release kinetics of drugs in PLGA NPs can be easily controlled by adjusting the characteristics of the polymer, such as the molecular weight and ratio of lactide and glycolide (LG ratio) [12,13,14]. Poly (lactic acid) (PLA) is a hydrophobic and stiff polymer, showing low mechanical strength. Compared to PLA, poly (glycolic acid) (PGA) is a hydrophilic and crystalline polymer, showing fast degradation with low water solubility. Thus, PLGA has intrinsic properties according to the polymeric content of LA and GA [15]. For example, the hydrophobicity of PLGA can increase by an increase in the LG ratio, resulting in reducing the degradation rate and showing slower drug release kinetics [16]. Furthermore, a higher GA content leads to a faster degradation, with an exception of the 50:50 LG ratio, which exhibits the fastest degradation [14]. The molecular weight of PLGA also affects the degradation and drug-release kinetics, wherein the decrease in molecular weight increase both the degradation rate and drug-release rate [17]. In addition, the degradation and drug-release kinetics of PLGA can be differ from the size of the resulting PLGA formulations.

Based on the intrinsic properties of PLGA, various PLGA formulations were used for cancer immunotherapy. For example, oxaliplatin-loaded methoxy-poly(ethylene glycol) (mPEG)–PLGA, as an ICD inducer, results in increasing the tumor-infiltrating CTLs after treatment with the oxaliplatin PLGA NPs [18]. In combination with an adjuvant, CpG oligonucleotide, ICD inducer-loaded PLGA particles can enhance the antitumor immune response. The CpG and DOX-loaded PLGA particles successfully reduced the progression of T-cell lymphoma as well as melanoma, resulting in showing potential for in situ immunization against tumor cells [19]. For effective cancer immunotherapy, the timing of the treatment of the ICD-inducers and immunotherapies can provide an impact on the therapeutic efficacy. In this point of view, sustained-release with single-inoculation therapy with PLGA NPs might provide advantages for priming and boosting of immune responses against tumors [20]. However, the release kinetics of the ICD-inducers from PLGA NPs, as well as their influence on the immune responses, are still not exemplified.

Herein, we hypothesized that the different release kinetics of DOX from different molecular weight PLGA-based NPs might induce the ICD of the tumor. Furthermore, the difference in ICD of the tumor might be related to the antitumor response after direct local delivery to the tumor site. To demonstrate our hypothesis, DOX was encapsulated with the different molecular weight of PLGA (7000 g/mol and 12,000 g/mol) by using the oil-in-water (o/w) emulsion method, resulting in preparing DOX-PLGA_7K_ NPs and DOX-PLGA_12K_ NPs. To confirm the in vitro characteristics of both the DOX-PLGA_7K_ NPs and DOX-PLGA_12K_ NPs, the morphology, drug loading efficiency, size distribution, surface charge, and stability were measured using field-emission scanning electron microscopy (FE-SEM), UV-Vis spectrometry, and a zeta-sizer, respectively. DOX release kinetics from DOX-PLGA_7K_ NPs and DOX-PLGA_12K_ NPs were monitored for 30 days under physiological conditions. The cellular uptake and cytotoxicity were also monitored. Subsequently, ICD of the CT-26 tumor cells was analyzed by observing HMGB1 release into the culture medium. To confirm the DOX release kinetics after direct tumor injection, we monitored the DOX fluorescence changes in the tumor tissue of the CT-26 tumor-bearing mice after direct tumor injection of the DOX-HCl, DOX-PLGA_7K_ NPs, and DOX-PLGA_12K_ NPs. Furthermore, tumor growth inhibition and immune cell changes were observed. Finally, the immunological memory effect of the tumor-suppressed mice was evaluated by monitoring tumor growth after re-challenging of the CT-26 tumor cells.

## 2. Materials and Methods 

### 2.1. Materials

Acid-terminated poly(lactic-*co*-glycolic acid) (PLGA, LA:GA = 50:50, 5000–10,000 g/mol (PLGA 7K)) was purchased from PolySciTech (Akina. Inc., West Lafayette, IN, USA). Acid-terminated PLGA (LA:GA = 50:50, 7000–17,000 g/mol (PLGA 12K)), poly(vinyl alcohol) (PVA, 87–90% hydrolyzed, 30,000–70,000 g/mol), fluorescein amine, triethylamine (TEA, 99%), and anhydrous dichloromethane (DCM) were purchased from Sigma-Aldrich (St. Luis, MO, USA). Doxorubicin hydrochloride (DOX-HCl) was purchased from FutureChem Co., Ltd. (Seoul, Korea). Rat anti-mouse CD16/CD32 (mouse BD Fc block^TM^) was purchased from BD Pharmingen^TM^ (BD Biosciences, San Jose, CA, USA). Phycoeythrin (PE)/Cyanine7-conjugated anti-mouse CD45.2 antibody, fluorescein isothiocyanate (FITC)-conjugated anti-mouse CD3 antibody, allophycocyanin (APC)-conjugated anti-mouse CD8a antibody, APC-conjugated anti-mouse CD11c antibody, PE-conjugated anti-mouse CD40 antibody, and FITC-conjugated anti-mouse CD86 antibody were purchased from BioLegend (San Diego, CA, USA). Mouse IFN-γ Quantikine ELISA Kit was purchased from R&D Systems, Inc. (Minneapolis, MN, USA). Mouse tumor dissociation kit was purchased from Miltenyi Biotec (Bergisch Gladbach, Germany). The mouse colon carcinoma cell CT-26 was purchased from the Korean Cell Line Bank (KCLB, Seoul, Korea). Fetal bovine serum, RPMI-1640 media, and antibiotics solution (1% penicillin and streptomycin) were purchased from Welgene Inc. (Gyeonsangbuk-do, Korea). All other chemicals were purchased as reagent grade and used without further purification.

### 2.2. Preparation of DOX-Loaded PLGA NPs

Before encapsulation of DOX into the PLGA NPs, 54.4 mg of DOX-HCl (100 µmole) was desalted using 200 µL of TEA in methanol (10 mL) for 6 h. The desalted DOX was extracted using chloroform and then was evaporated under reduced pressure. Two kinds of DOX-loaded PLGA NPs were prepared using acid-terminated PLGA 7K and PLGA 12K by the oil-in-water (o/w) single emulsion method [21]. In brief, 20 mg of DOX was dissolved in 0.48 mL of DCM. Then, 20 mg of PLGA 7K or PLGA 12K was dissolved in 1 mL of DCM, followed by mixing with 0.24 mL of the DOX solution, respectively. The mixtures were emulsified in 6 mL of aqueous PVA solution (3 wt%) by sonication for 1 min (180 watts) in an ice bath. These emulsions were then diluted in 20 mL of a PVA solution (0.3 wt%), followed by stirring for 3 h at room temperature to remove the DCM. Then, both PLGA NPs were washed two times with distilled water by centrifugation at 15,000 rpm for 20 min. Finally, the resulting suspensions were lyophilized to obtain the DOX-loaded PLGA 7K NPs (DOX-PLGA_7K_ NPs) and DOX-loaded PLGA 12K NPs (DOX-PLGA_12K_ NPs). As the control PLGA NPs, PLGA_7K_ NPs, and PLGA_12K_ NPs were formulated by an oil-in-water single emulsion method without DOX. For the preparation of fluorescent dye-labeled PLGA NPs, both acid-terminated PLGA 7K and acid-terminated PLGA 12K were chemically conjugated with fluorescein amine in the presence of N-(3-Dimethylaminopropyl)-N′-ethylcarbodiimide (EDC) hydrochloride and N-hydroxysuccinimide (NHS). In brief, 10 mg of PLGA 7K or 20 mg of PLGA 12K was dissolved in 5 mL of dimethylsulfoxide (DMSO), followed by mixing with 1 mL of DMSO containing 550 µg of EDC and 330 µg of NHS. Then, 1 mL of fluorescein amine solution (2 mg/mL in DMSO) was dropwise added into each PLGA solution and vigorously stirred for 12 h at room temperature. The mixtures were then purified with a dialysis membrane (MW cut-off 3500 Da, Spectra/Por^®^ 3 Dialysis membrane, Repligen Corporation, Waltham, MA, USA) against DMSO:distilled water (100:0, 50:50, and 0:100 *v/v*%) for 3 days. The resulting solutions were lyophilized to obtain FITC-PLGA_7K_ and FITC-PLGA_12K_.

### 2.3. In Vitro Characterization of DOX-PLGA_7K_ NPs and DOX-PLGA_12K_ NPs

The hydrodynamic diameter, size distribution, and surface charge of the PLGA_7K_ NPs, PLGA_12K_ NPs, DOX-PLGA_7K_ NPs, and DOX-PLGA_12K_ NPs, which were freshly dispersed in the distilled water (0.2 mg/mL), were measured using a Zetasizer Nano-ZS (Malvern Instruments, London, UK). The morphology of the PLGA_7K_ NPs, PLGA_12K_ NPs, DOX-PLGA_7K_ NPs, and DOX-PLGA_12K_ NPs was observed using field emission scanning electron microscopy (Nova Nano SEM 200, FEI Company, Hillsboro, OR, USA). The DOX-loading efficiency of DOX-PLGA_7K_ NPs and DOX-PLGA_12K_ NPs were determined using UV-Vis spectrometry (Agilent Cary 60 UV-Vis spectrophotometer, Agilent Technologies, Santa Clara, CA, USA) by measuring the absorbance at 480 nm. In brief, 1 mg of DOX-PLGA_7K_ NPs or DOX-PLGA_12K_ NPs was dissolved in 1 mL of dimethylsulfoxide (DMSO). The baseline was corrected using 1 mL of DMSO before measurement. Then, the absorbance of the DOX-PLGA_7K_ NPs or DOX-PLGA_12K_ NPs at 480 nm was analyzed based on a standard curve, which was measured at a predetermined concentration of DOX-HCl. The size stability of the DOX-PLGA_7K_ NPs and DOX-PLGA_12K_ NPs was monitored using a Zetasizer Nano-ZS for 30 days. In brief, 1 mg of DOX-PLGA_7K_ NPs or DOX-PLGA_12K_ NPs was dispersed in phosphate-buffered saline (PBS, pH 7.4) containing 10% (*v/v*) of FBS, followed by incubating at 37 °C for 30 days. The size of both the DOX-PLGA_7K_ NPs and DOX-PLGA_12K_ NPs was recorded every 2 days using a Zetasizer Nano-ZS. The in vitro DOX release profiles of the DOX-PLGA_7K_ NPs and DOX-PLGA_12K_ NPs were monitored in PBS (pH 7.4) containing 0.1% Tween 80 at 37 °C for 30 days. In brief, 4 mg of the DOX-PLGA_7K_ NPs (300 µg of DOX) and 8 mg of the DOX-PLGA_12K_ NPs (300 µg of DOX) were dispersed in 4 mL of medium. A 1 mL sample of each was transferred into the dialysis membrane (MW cut-off = 12–14 KDa, Spectra/Por^®^ 4 Dialysis membrane, Repligen Corporation, Waltham, MA, USA) and was incubated at 37 °C in a shaking water bath. The medium was changed with 20 mL of fresh medium at the pre-determined time points. The amount of released DOX was analyzed using the UV-Vis spectrometer (Agilent Cary 60 UV-Vis spectrophotometer, Agilent Technologies, Santa Clara, CA, USA) by measuring absorbance at 480 nm.

### 2.4. In Vitro Cellular Uptake, Cytotoxicity, and Immunogenic Cell Death Analysis

Prior to observing cellular uptake, 10 wt% of FITC-PLGA_7K_ and FITC-PLGA_12K_ were used for formulating the DOX-PLGA_7K_ NPs and DOX-PLGA_12K_ NPs, respectively. In vitro cellular uptake of the DOX-PLGA_7K_ NPs and DOX-PLGA_12K_ NPs was observed in the CT-26 tumor cells by using a confocal laser scanning microscope (CLSM). In brief, CT-26 tumor cells (5 × 10^4^ cells) were seeded into a 35-mm confocal dish and stabilized for 24 h. The CT-26 tumor cells were then treated with RPMI 1640 media containing DOX-PLGA_7K_ NPs, DOX-PLGA_12K_ NPs (2 µM of DOX), or 2 µM of DOX-HCl for 48 h at 37 °C. Then, the CT-26 tumor cells were washed three times with DPBS (pH 7.4) and fixed with a 4% paraformaldehyde solution for 15 min. The nuclei of the CT-26 tumor cells were stained with DAPI for 5 min at room temperature, followed by the fluorescence signals being observed using a CLSM (Leica TCS SP8, Wetzlar, Germany) equipped with a 405 diode (405 nm), Ar lasers (488, 514 nm), and HeNe-Red (633 nm) lasers.

The cytotoxic effect of the DOX-PLGA_7K_ NPs and DOX-PLGA_12K_ NPs in the CT-26 tumor cells were evaluated using a CCK-8 assay kit. In brief, 5 × 10^3^ of the CT-26 tumor cells were seeded onto a 96-well plate and stabilized for 24 h. Then, CT-26 tumor cells were treated with DOX-HCl, DOX-PLGA_7K_ NPs, and DOX-PLGA_12K_ NPs (0.001 to 10 µM of DOX) for 48 h, followed by further being incubated with a 10% (*v/v*) CCK-8 solution containing RPMI1640 medium for 1 h. Finally, the absorbance of the medium was analyzed at 450 nm using a microplate reader (VERSAmaxTM, Molecular Devices Corp., San Jose, CA, USA). As a control, the cell viability of the CT-26 tumor cells, which were treated with 0.1, 1, 10, and 100 µg/mL of PLGA_7K_ NPs or PLGA_12K_ NPs, was measured using the same CCK-8 assay.

To observe the DOX-induced ICD in CT-26 tumor cells in vitro, HMGB1 was selected as a molecular marker of ICD and the released HMGB1 in the cell culture medium was analyzed using Western blot. In brief, 1 × 10^6^ of the CT-26 tumor cells were seeded onto 6-well cell culture plates and stabilized for 24 h. Then the DOX-HCl, DOX-PLGA_7K_ NPs, DOX-PLGA_12K_ NPs (5 µM of DOX), and PLGA_7K_ NPs were treated for 48 h. Subsequently, the culture medium was collected. The culture medium was centrifuged for 12 min at 1200 rpm to remove cell debris. Then, the supernatant was concentrated up to 0.5 mL using centrifugal filter units (Amicon^®^ Ultra, MW cut-off 10 KDa). Each sample was mixed with sodium dodecyl sulfate (SDS) gel-loading buffer (125 mol/L Tris, pH 6.8, 5% glycerol, 2% SDS, 1% mercaptoethanol, and 0.006% bromophenol blue) and boiled for 5 min. Then, 20 µL of the samples were separated by 10% SDS-polyacrylamide gel electrophoresis and transferred onto a nitrocellulose membrane. The membranes were blocked for 1 h at room temperature in 5% bovine serum albumin (BSA) containing a 1 × TBST solution (10 mol/L Tris, pH 7.4, 100 mol/L NaCl, and 0.1% Tween 20). Then the membranes were incubated with rabbit polyclonal HMGB1 antibody (1:500, abcam, Cambridge, MA, USA) for 12 h at 4 °C. The membranes were washed 3 times and incubated with goat anti-rabbit IgG-HRP antibody (1:1000, abcam, USA) for 1 h at room temperature. After 3 more washes with 1× TBST, the HMGB1 protein band was detected with an ECL system. The HMGB1 band intensities were observed using ImageJ software (NIH, Bethesda, MD, USA).

### 2.5. In Vivo Drug Release Analysis

All experiments with live animals were performed in compliance with the relevant laws and institutional guidelines of the Korea Institute of Science and Technology (KIST), and institutional committees approved the experiments (approval number: KIST-2020-070, 27/05/2020). The DOX release from DOX-PLGA_7K_ NPs and DOX-PLGA_12K_ NPs was observed by monitoring DOX fluorescence in CT-26 tumor-bearing Balb/c mouse after intratumoral injection. In brief, 2 × 10^6^ of the CT-26 tumor cells were subcutaneously injected on the left flank of a 6-week-old male Balb/c mouse to establish the tumor-bearing mouse model. Then, DOX-HCl, DOX-PLGA_7K_ NPs, or DOX-PLGA_12K_ NPs (10 mg/kg of DOX per mice) in 30 μL of PBS were directly injected into the tumor tissue when the tumor volume reached to 65 ± 10 mm^3^ (*n* = 3 per group). The fluorescence signals from the DOX in the tumor tissues were monitored for 17 days using a small animal fluorescence imaging system (IVIS-Lumina III In Vivo Imaging System, Perkin Elmer, Waltham, MA, USA) and quantified using Living Image Software 4.0 (Perkin Elmer, MA, USA). To monitor the DOX fluorescence in the major organs and tumor tissue, the CT-26 tumor-bearing mice were sacrificed at 17 days post-injection, followed by the fluorescence signals from the liver, lung, kidney, spleen, heart, and tumor tissue being measured using an IVIS-Lumina III In Vivo Imaging System. The DOX fluorescence from the excised tumor tissues was quantified using Living Image Software 4.0.

### 2.6. Tumor Growth Inhibition and Mechanism Analysis

The tumor growth inhibition effect and mechanism analysis were performed in the CT-26 tumor-bearing mice after direct tumor injection of DOX-HCl, DOX-PLGA_7K_ NPs, and DOX-PLGA_12K_ NPs. First, tumor tissue morphology was observed using Hematoxylin and Eosin (H&E) staining to confirm the DOX-induced cell death on 14 days after treatment of DOX-HCl, DOX-PLGA_7K_ NPs, and DOX-PLGA_12K_ NPs. In brief, 1 × 10^6^ of CT-26 tumor cells were subcutaneously injected on the left flank of a 6-week-old male Balb/c mouse to establish a tumor-bearing mouse model. Then, 30 μL of PBS (*n* = 3), PLGA_7K_ NPs (*n* = 3), DOX-HCl (*n* = 3), DOX-PLGA_7K_ NPs (*n* = 3), or DOX-PLGA_12K_ NPs (*n* = 3) (10 mg/kg of DOX per mice) in 30 μL of PBS were directly injected to tumor tissue when the tumor volume reached to 65 ± 10 mm^3^. Tumor tissues were obtained after 14 days post-treatment, and the tumor tissues were fixed with 4% paraformaldehyde solution, followed by embedding in paraffin after dehydration. Tumor tissue slices of 10 μm were stained with Hematoxylin and Eosin (H&E).

Secondly, dendritic cells (DCs) and cytotoxic T-lymphocytes (CTLs) were analyzed using isolated tumor-draining lymph nodes (TDLNs) and tumor tissues from the CT-26 tumor-bearing mice, respectively. 1 × 10^6^ of CT-26 tumor cells were subcutaneously injected on the left flank of a 6-week-old male Balb/c mouse. Then, DOX-HCl (*n* = 5), DOX-PLGA_7K_ NPs (*n* = 5), or DOX-PLGA_12K_ NPs (*n* = 5) (10 mg/kg of DOX per mice) in 30 μL of PBS were directly injected into the tumor tissue when the tumor volume reached to 65 ± 10 mm^3^. As a control, PLGA_7K_ NPs (*n* = 5), which showed a faster DOX-release kinetic than PLGA_12K_ NPs, were directly injected into the tumor tissue to monitor the changes in DCs and CTLs. Tumor tissues and TDLNs were obtained after 14 days post-treatment, followed by cutting into small pieces and incubated with digestive enzymes at 2% FBS containing DPBS (pH 7.4). Then, red blood cells (RBCs) were removed using RBC lysis buffer, followed by the single-cell suspension that was stained with fluorescent dye-conjugated antibodies. Total DCs, maturated DCs, and CTLs were stained with anti-CD45.2 and anti-CD11c (total DCs), anti-CD45.2, anti-CD11c, anti-CD40, and anti-CD86 (maturated DCs), and anti-CD45.2, anti-CD3, and anti-CD8 antibodies, and then analyzed using flow cytometry (BD Accuri C6 Flow Cytometer, BD Biosciences, NJ, USA). All these antibodies were freshly diluted (~200 times) in 2% FBS containing DPBS (pH 7.4) before staining of the cells. Total DCs, maturated DCs, and CTLs were analyzed using FlowJo^TM^ software (Becton, Dickinson and Company, Franklin Lakes, New Jersey, USA) wherein the population of total DCs, maturated DCs, and CTLs were CD45.2^+^ CD11c^+^, CD40^+^ CD86^+^, and CD45.2^+^ CD3^+^ CD8^+^, respectively.

Finally, tumor growth was monitored for 28 days and tumor-suppressed mice was selected based on tumor volume (tumor volume < 300 mm^3^) at 28 days after treatment. In brief, 1 × 10^6^ of CT-26 tumor cells were subcutaneously injected on the left flank of a 6-week-old male Balb/c mouse to establish the tumor-bearing mouse model. DOX-PLGA_7K_ NPs (*n* = 13) or DOX-PLGA_12K_ NPs (*n* = 13) (10 mg/kg of DOX per mice) in 30 μL of PBS were directly injected into the tumor tissue when the tumor volume reached 65 ± 10 mm^3^. As a control group, 30 μL of PBS (*n* = 5) and DOX-HCl (*n* = 13, 10 mg/kg) were directly injected into the tumor tissue. The survival of the CT-26 tumor-bearing mice were recorded for 28 days, wherein mice with a tumor size of 2000 mm^3^ or more before day 28 were counted as dead. The tumor volume was calculated using the following formula: V = 0.52 × L × W^2^ (longitudinal diameter (L) and transverse diameter (W)).

### 2.7. Tumor Re-Challenging and Interferon-Gamma (INF-γ) Detection

To confirm the long-term immune memory effect in the mice, 1 × 10^6^ of the CT-26 tumor cells were re-challenged into the right flank of the tumor-suppressed Balb/c mice on 28 days post-treatment of DOX-HCl (*n* = 7), DOX-PLGA_7K_ NPs (*n* = 7) or DOX-PLGA_12K_ NPs (*n* = 4). The tumor volume was monitored for 18 days and was calculated using the following formula: V = 0.52 × L × W^2^ (longitudinal diameter (L) and transverse diameter (W)). The amount of INF-γ in the serum samples, which were isolated from the mice, were analyzed with an INF-γ ELISA Kit according to the manufacturer’s protocols. 

### 2.8. Statistical Analysis

In this study, the differences between the experimental and control groups were analyzed through one-way ANOVA using Origin 2020 software (OriginLab Corporation, Northampton, MA, USA) and considered statistically significant when marked with an asterisk (*) in the figure.

## 3. Results and Discussions

### 3.1. Formulation and In Vitro Characterization of the DOX-PLGA_7K_ NPs and DOX-PLGA_12K_ NPs

**Figure 1:**
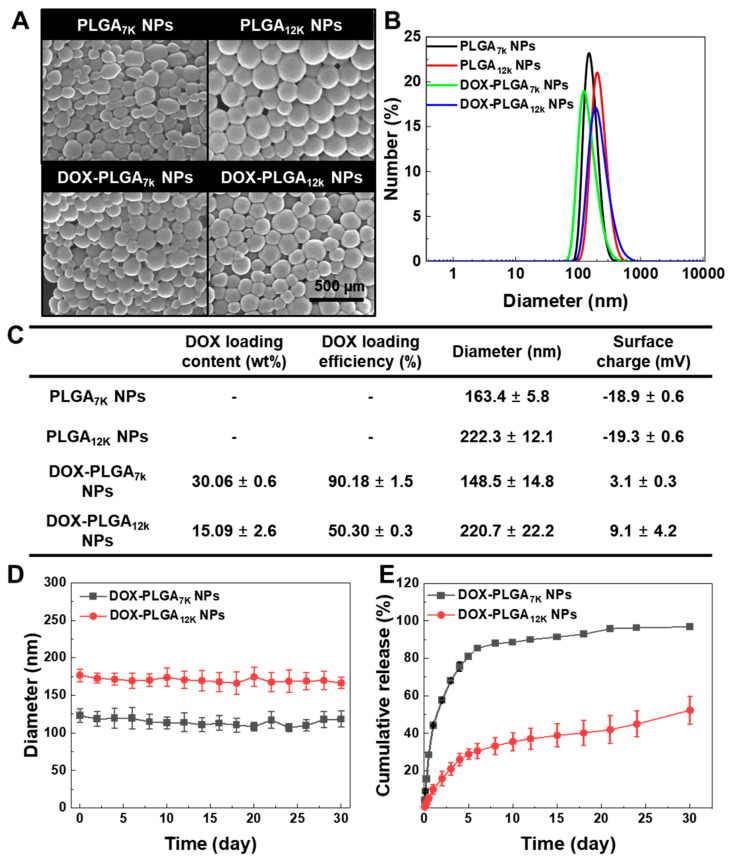
In vitro physicochemical properties of the PLGA nanoparticles (NPs). The morphologies (**A**) and size distribution (**B**) of the PLGA_5K_ NPs, PLGA_12K_ NPs, DOX-PLGA_7K_ NPs, and DOX-PLGA_12K_ NPs were observed using FE-SEM and a zeta-sizer, respectively. The size, surface charge, DOX loading content, and DOX loading efficiency were summarized in the characteristics table (**C**). (**D**) The size stability of the DOX-PLGA_7K_ NPs and DOX-PLGA_12K_ NPs for 30 days. (**E**) In vitro DOX release of the DOX-PLGA_7K_ NPs and DOX-PLGA_12K_ NPs. When DOX was loaded into the PLGA NPs, the DOX release kinetics were changed by the molecular weight of the PLGA NPs, resulting in the DOX release from the DOX-PLGA_7K_ NPs being relatively faster than from the DOX-PLGA_12K_ NPs.

In this study, we prepared DOX-PLGA_7K_ NPs and DOX-PLGA_12K_ NPs by using the single o/w emulsion method to compare their therapeutic potential based on their drug release behavior. The amount of DOX in 1 mg of DOX-PLGA_7K_ NPs and DOX-PLGA_12K_ NPs was measured by a UV-Vis spectrophotometer, wherein 300.6 ± 6 μg and 150.9 ± 26 μg of DOX were encapsulated, respectively. The difference in DOX content between the DOX-PLGA_7K_ NPs and DOX-PLGA_12K_ NPs would be affected by the difference in the solubility and miscibility of the polymers, DOX, or solution (distilled water, DCM, PVA) during the formulation [22]. DOX-PLGA_7K_ NPs and DOX-PLGA_12K_ NPs were then characterized to observe their morphology, size, and surface charge in the physiological condition. PLGA_7K_ NPs and PLGA_12K_ NPs were used as the control NPs. Based on the FE-SEM images, the DOX-PLGA_7K_ NPs and DOX-PLGA_12K_ NPs were spherical with a smooth surface, similar to those of the PLGA_7K_ NPs and PLGA_12K_ NPs (Figure 1A). Furthermore, the DOX-PLGA_7K_ NPs and DOX-PLGA_12K_ NPs as well as PLGA_7K_ NPs and PLGA_12K_ NPs exhibited a unimodal size distribution, wherein the average diameter of the DOX-PLGA_7K_ NPs, DOX-PLGA_12K_ NPs, PLGA_7K_ NPs, and PLGA_12K_ NPs was 148.5 ± 14.8, 220.7 ± 22.2, 163.4 ± 5.8, and 222.3 ± 12.1 nm, respectively (Figure 1B). The surface charge of the PLGA_7K_ NPs, PLGA_12K_ NPs, DOX-PLGA_7K_ NPs, and DOX-PLGA_12K_ NPs were −18.9 ± 0.6, −19.3 ± 0.6, 3.1 ± 0.3, and 9.1 ± 4.2 mV, respectively, resulting in a changing surface charge after DOX encapsulation. This is because that cationic drug DOX might interact with the anionic surface of the PLGA NPs as well as encapsulating into the hydrophobic inner space of the PLGA NPs [23]. The characteristic values of the PLGA_7K_ NPs, PLGA_12K_ NPs, DOX-PLGA_7K_ NPs, and DOX-PLGA_12K_ NPs were summarized in Figure 1C. The size of the DOX-PLGA_7K_ NPs and DOX-PLGA_12K_ NPs were stable in 10% FBS-contained PBS for 30 days (Figure 1D). Next, we measured the DOX release profiles of the DOX-PLGA_7K_ NPs and DOX-PLGA_12K_ NPs for 30 days. Both the DOX-PLGA_7K_ NPs and DOX-PLGA_12K_ NPs were dispersed in PBS (pH 7.4, 0.1% tween 80) and incubated in a shaking water bath at 37 °C. As a result, the DOX-PLGA_7K_ NPs and DOX-PLGA_12K_ NPs showed different release kinetics for 30 days under physiological conditions. A total of 96.7 ± 0.8% and 52.2 ± 7.4% of DOX was released from the DOX-PLGA_7K_ NPs and DOX-PLGA_12K_ NPs for 30 days. On Day 30, the remaining DOX molecules in the DOX-PLGA_7K_ NPs and DOX-PLGA_12K_ NPs were measured as 2.4 ± 0.3% and 47.1 ± 1.8%, based on the feed ratio of DOX in the PLGA NPs. After 5 days, in particular, 80.9% and 28.9% of the DOX was released from the DOX-PLGA_7K_ NPs and DOX-PLGA_12K_ NPs, respectively, showing that the DOX-PLGA_7K_ NPs had an initial bust release and a faster DOX release profile than the DOX-PLGA_12K_ NPs. This results were correlated with other previous reports that the decrease in molecular weight of the PLGA was increasing the degradation rate, resulting in promoting drug release [12,24]. Based on these results, the DOX-PLGA_7K_ NPs and DOX-PLGA_12K_ NPs were successfully formulated, resulting in forming stable nano-sized spherical particles. However, the DOX-PLGA_7K_ NPs and DOX-PLGA_12K_ NPs showed a different NP size, surface charge, and DOX loading content, resulting in showing different DOX release kinetics in vitro.

### 3.2. In Vitro Cellular Uptake and Immunogenic Cell Death on the CT-26 Tumor Cells

**Figure 2:**
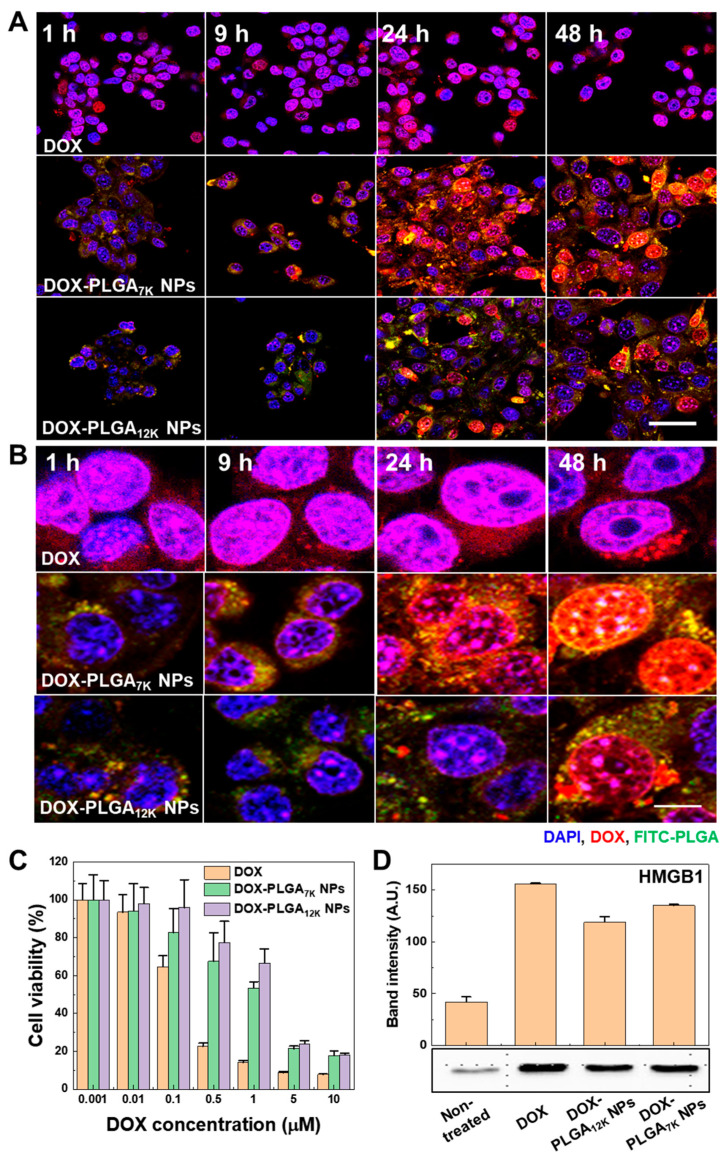
In vitro cellular uptake and immunogenic cell death on the CT-26 tumor cells. (**A**) Time-dependent cellular uptake of the DOX-HCl, FITC-labeled DOX-PLGA_7K_ NPs, and FITC-labeled DOX-PLGA_12K_ NPs. Blue channel: DAPI; Red channel: DOX; Green channel: FITC-labeled PLGA NPs. The scale bar indicates 20 μm. (**B**) Magnified fluorescence images of the DOX-HCl-, FITC-labeled DOX-PLGA_7K_ NP-, and FITC-labeled DOX-PLGA_12K_ NP-treated CT-26 tumor cells. Blue channel: DAPI; Red channel: DOX; Green channel: FITC-labeled PLGA NPs. The scale bar indicates 5 μm. (**C**) The cell viability of the CT-26 tumor cells treated with various concentrations (0.001 to 10 µM of DOX) of DOX-HCl, DOX-PLGA_7K_ NPs, or DOX-PLGA_12K_ NPs for 48 h. (**D**) Western blot image and band intensity of HMGB1, which was released in the cell culture medium. CT-26 tumor cells were treated with DOX-HCl, DOX-PLGA_7K_ NPs, or DOX-PLGA_12K_ NPs (5 µM of DOX) for 48 h.

To observe intracellular delivery of DOX, and DOX-induced cytotoxicity with ICD on the CT-26 tumor cells, fluorescence imaging, cytotoxicity, and HMGB1 release analysis were performed by using CLSM, CCK-8, and Western blot analysis, respectively. First, CT-26 tumor cells were treated with DOX-HCl, FITC-labeled DOX-PLGA_7K_ NPs, or FITC-labeled DOX-PLGA_12K_ NPs (2 μM of DOX) for 1 h, 9 h, 24 h, and 48 h, followed by observing the fluorescence signals of FITC and DOX. The CLSM images showed that the green fluorescence signals from both the DOX-PLGA_7K_ NPs and DOX-PLGA_12K_ NPs were located in the cytoplasm of the CT-26 tumor cells, resulting in an increasing fluorescence signal by the incubation time (Figure 2A). Magnified fluorescence images showed that DOX fluorescence of the DOX-HCl-treated CT-26 tumor cells was mainly observed in the nucleus at 1 h whereas the DOX-PLGA_7K_ NP-treated CT-26 tumor cells, mainly observed in the cytoplasm at 1 h, resulted in gradually localizing at the nucleus at 24 h and 48 h (Figure 2B). Furthermore, the DOX-PLGA_12K_ NP-treated CT-26 tumor cells showed that DOX localization in the nucleus was mainly observed at 48 h (Supplementary Materials Figure S1). The difference in DOX localization into the nucleus of the CT-26 tumor cells would have occurred due to the different DOX release kinetics from the DOX-PLGA_7K_ NPs and DOX-PLGA_12K_ NPs, wherein a faster release of DOX from the DOX-PLGA_7K_ NPs showed faster DOX localization in the nucleus compared to that of the DOX-PLGA_12K_ NPs. Next, DOX-induced cytotoxicity on the CT-26 tumor cells was measured using CCK-8 after treatment of various doses of DOX-HCl, DOX-PLGA_7K_ NPs, or DOX-PLGA_12K_ NPs (0.001 to 10 µM of DOX) for 48 h (Figure 2C). The cell viability of the DOX-HCl-, DOX-PLGA_7K_ NPs-, or DOX-PLGA_12K_ NP-treated CT-26 tumor cells was gradually decreased by the treatment concentration manner. Furthermore, the half-maximal inhibitory concentration (IC50) values were 0.237 µM, 1.32 µM, and 2.22 µM when the cells were treated with DOX-HCl, DOX-PLGA_7K_ NPs, and DOX-PLGA_12K_ NPs, respectively. Since DOX can activate cytotoxicity after localization at the nucleus, the DOX-PLGA_7K_ NPs, which exhibited a faster DOX release and nucleus localization than the DOX-PLGA_12K_ NPs, were more efficient in killing the CT-26 tumor cells than the DOX-PLGA_12K_ NPs. However, sustained-release profiles of DOX from the DOX-PLGA_7K_ NPs and DOX-PLGA_12K_ NPs reduced the cytotoxic effect on the CT-26 tumor cells compared to DOX-HCl. As a control, the PGLA_7K_ NPs and PLGA_12K_ NPs showed no significant cytotoxic effect on the CT-26 tumor cells. When the CT-26 tumor cells were treated with 100 µg/mL of PGLA_7K_ NPs and PLGA_12K_ NPs for 48 h, the cell viability was measured as 94.5 ± 6.0% and 99.9 ± 7.3%, respectively, compared to the non-treated CT-26 tumor cells (Supplementary Materials Figure S2A). Finally, we observed HMGB1 release from the CT-26 tumor cells to analyze the DOX-induced ICD on the CT-26 tumor cells. HMGB1 is passively released protein from dying cells by ICD [25]. Notably, HMGB1 can engage with TLR-4 and a receptor for advanced glycation end-products on DCs, resulting in eliciting proinflammatory cytokine secretion and promoting cross-presentation of tumor antigens to T lymphocytes [26,27]. The released HMGB1 from the CT-26 tumor cells, which were treated with DOX-HCl, DOX-PLGA_7K_ NPs, and DOX-PLGA_12K_ NPs for 48 h, was analyzed using Western blot. Figure 2D showed that the HMGB1 band intensity was significantly increased when the CT-26 tumor cells were treated with DOX-HCl, DOX-PLGA_7K_ NPs, and DOX-PLGA_12K_ NPs, compared to that of non-treated CT-26 tumor cells. Furthermore, the relative HMGB1 band intensities of the DOX-HCl-, DOX-PLGA_7K_ NP-, and DOX-PLGA_12K_ NP-treated CT-26 tumor cells were 3.70-, 3.21-, and 2.80-fold higher than that of the non-treated CT-26 tumor cells, respectively, correlating with their cell viability profile after treatment. As a control experiment, PLGA_7K_ NP-treated CT-26 tumor cells showed no significant difference in HMGB1 release compared to that of the non-treated CT-tumor cells (Supplementary Materials Figure S2B). These results showed that the DOX-PLGA_7K_ NPs and DOX-PLGA_12K_ NPs could successfully induce ICD to CT-26 tumor cells via the sustained release of DOX to the CT-26 tumor cells.

### 3.3. In Vivo Drug Release and Tumor Growth Inhibition

**Figure 3:**
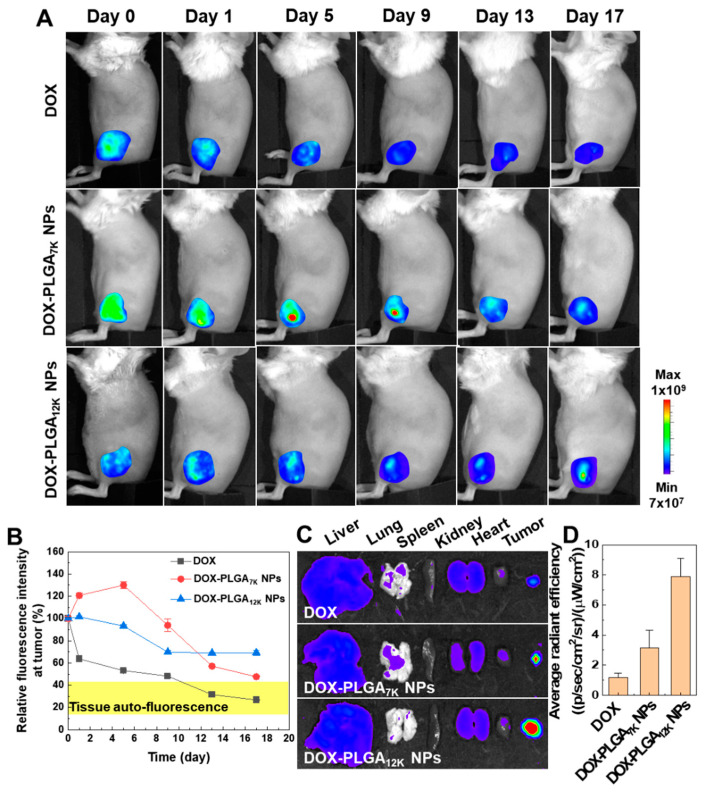
In vivo drug release of DOX-PLGA_7K_ NPs or DOX-PLGA_12K_ NPs after single direct-injection at the tumor. (**A**) Whole-body DOX fluorescence images of a CT-26 tumor-bearing mouse injected with DOX-HCl, DOX-PLGA_7K_ NPs, or DOX-PLGA_12K_ NPs. (**B**) Quantified relative DOX fluorescence intensity at the tumor tissue based on the fluorescence images from (**A**). (**C**) The representative ex vivo DOX fluorescence images of the DOX-HCl-, DOX-PLGA_7K_ NP-, or DOX-PLGA_12K_ NP-treated mouse on Day 17. (**D**) The quantified average DOX fluorescence signals at the tumor tissues based on the NIRF images from (**C**).

To observe DOX release from DOX-PLGA_7K_ NPs and DOX-PLGA_12K_ NPs in vivo, the DOX fluorescence signals in tumor tissues were directly visualized using an In Vivo Imaging System. Because the NIRF intensity of the free DOX is not large enough in vivo, there is no difference from the comparable auto-fluorescence signals in whole-body in saline-treated mice (Supplementary Materials Figure S3). After direct tumoral injection of DOX-HCl (10 mg/kg), DOX-PLGA_7K_ NPs (10 mg/kg of DOX), or DOX-PLGA_12K_ NPs (10 mg/kg of DOX), the fluorescence signals of DOX at the tumor tissues were monitored for 17 days. The DOX-HCl-treated CT-26 tumor tissue showed that visible DOX fluorescence at Day 0 was drastically decreased compared to Day 9, resulting in showing no significant difference with tissue auto-fluorescence on Day 9 (Figure 3A). However, the DOX fluorescence signals of the DOX-PLGA_7K_ NP- or DOX-PLGA_12K_ NP-treated tumor tissues were maintained for 17 days, showing a significantly higher fluorescence signal in the tumor tissue than the surrounding tissue. The relative fluorescence intensity of the DOX-HCl-, DOX-PLGA_7K_ NP-, or DOX-PLGA_12K_ NP-treated CT-26 tumor tissues showed different patterns that were continuously decreased, drastically decreased after initial ascending, or with sustained fluorescence, respectively (Figure 3B). As expected, DOX fluorescence was mainly observed at the tumor tissue compared to the major organs (liver, lung, spleen, kidney, and heart), wherein strong DOX fluorescence showed in the order of DOX-HCl-, DOX-PLGA_7K_ NP-, or DOX-PLGA_12K_ NP-treated CT-26 tumor tissue (Figure 3C). As a result, DOX fluorescence of the DOX-PLGA_7K_ NP- or DOX-PLGA_12K_ NP-treated CT-26 tumor tissues was 2.63- and 6.62-fold higher than that of the DOX-HCl-treated CT-26 tumor tissues, respectively (Figure 3D). These results showed that both the PLGA NPs could change the pharmacokinetic profile of the directly injected DOX by sustained release of DOX to the tumor tissue.

**Figure 4:**
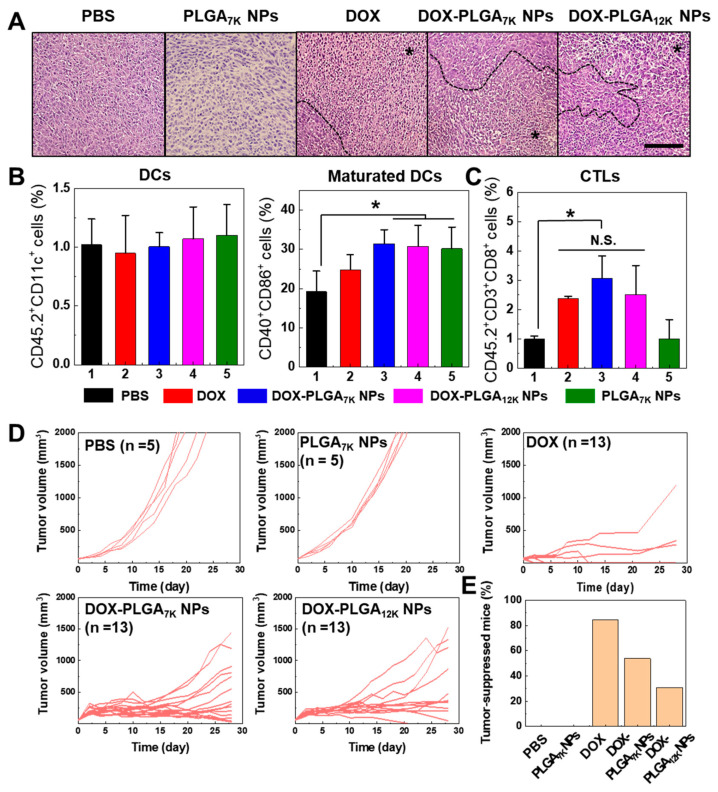
In vivo tumor growth inhibition and immune analysis. (**A**) H&E staining images of the CT-26 tumor tissue at 14 days after single direct-tumor injection of PBS, PLGA_7K_ NPs, DOX-HCl-, DOX-PLGA_7K_ NPs-, or DOX-PLGA_12K_ NPs (10 mg/kg of DOX). An asterisk (*) indicates a necrotic area in the H&E staining image. The scale bar indicates 200 μm. (**B**) Total DC populations and maturated DC populations in the TDLN and (**C**) CTL population in the CT-26 tumor tissues at 14 days after a single direct-tumor injection of PBS, PLGA_7K_ NPs, DOX-HCl-, DOX-PLGA_7K_ NPs-, or DOX-PLGA_12K_ NPs (10 mg/kg of DOX). Total DCs, maturated DCs, and CTLs were analyzed using a flow cytometer. An asterisk (*) indicates a difference at the *p* < 0.05 significance level. (**D**) Tumor growth of a CT-26 tumor-bearing mouse after single direct-tumor injection of PBS, PLGA_7K_ NPs, DOX-HCl-, DOX-PLGA_7K_ NPs-, or DOX-PLGA_12K_ NPs (10 mg/kg of DOX). (**E**) The ratio of the tumor-suppressed mouse after the PBS, PLGA_7K_ NP, DOX-HCl-, DOX-PLGA_7K_ NP, or DOX-PLGA_12K_ NP treatments.

Next, we confirmed the DOX-induced immune microenvironment changes and growth inhibition effect in the CT-26 tumor-bearing mice model. Prior to the animal experiment, we tested different doses and injection times of the DOX-HCl-, DOX-PLGA_7K_ NPs, or DOX-PLGA_12K_ NPs to decide the optimal dose of DOX. The DOX-HCl-, DOX-PLGA_7K_ NPs, and DOX-PLGA_12K_ NPs (5 mg/kg of DOX (single injection), 5 mg/kg of DOX (double injection), 10 mg/kg of DOX (single injection), and 10 mg/kg (double injection))-treated tumors were substantially inhibited when the tumor tissue was treated with 10 mg/kg of DOX-containing PLGA NPs. However, 10 mg/kg of DOX-HCl (double injection)-treated mice showed serious in vivo toxicity, such as body weight loss and normal tissue damages. Therefore, we selected the DOX concentration to 10 mg/kg with a single injection for further animal experiments. First, we observed tissue morphologies using H&E staining of DOX-HCl-, DOX-PLGA_7K_ NP-, or DOX-PLGA_12K_ NP-treated CT-26 tumor tissues at 14 days post single-direct injection. As a control group, the CT-26 tumor tissues were treated with PBS or PLGA_7K_ NPs. The CT-26 tumors of the PBS- and PLGA_7K_ NP-treated mice showed minimal necrotic area, indicating that there was no toxicity. In contrast, the DOX-HCl-treated tumors showed broad necrotic areas (indicated with an asterisk (*)) over the tumor tissues since the fast absorption and high toxicity of DOX-HCl. Furthermore, the DOX-PLGA_7K_ NP- or DOX-PLGA_12K_ NP-treated CT-26 tumor tissues included several large necrotic areas, resulting in exhibiting similar histological changes in the DOX-HCl-treated CT-26 tumor tissues (Figure 4A). The ICD in tumor tissue plays a key role in stimulating antitumor immune responses. In particular, maturation of immature DCs promotes phagocytic activity and engulfment of the antigenic component onto DCs, resulting in eliciting tumor cell-specific immune responses of T-cells via antigen presentation. Thus, DCs and CTLs were analyzed using isolated tumor-draining lymph nodes (TDLNs) and tumor tissues from the CT-26 tumor-bearing mice at 14 days post-direct injection of PBS, PLGA_7K_ NPs, DOX-HCl, DOX-PLGA_7K_ NPs, or DOX-PLGA_12K_ NPs. The DC population in the TDLNs showed no significant change between the PBS, PLGA_7K_ NPs, DOX-HCl-, DOX-PLGA_7K_ NP-, and DOX-PLGA_12K_ NP-treated CT-26 tumor-bearing mice. However, maturated DC populations in TDLNs in the PLGA_7K_ NPs, DOX-PLGA_7K_ NP-, and DOX-PLGA_12K_ NP-treated groups were significantly higher than that of the PBS-treated group (Figure 4B). The population of maturated DCs was increased when PLGA_7K_ NP-treated, showing a similar cell population to the DOX-loaded PLGA_7K_ NP- or DOX-loaded PLGA_12K_ NP-treated mice. This is because internalization by phagocytosis of PLGA NPs in immature DCs can induce DC maturation by an upregulation in CD86 expression [28]. However, the DOX-HCl-, DOX-PLGA_7K_ NP-, and DOX-PLGA_12K_ NP-treated groups showed significantly higher CTL populations in the tumor tissue than that of the PBS- or PLGA_7K_ NP-treated groups, indicating that the CTLs were successfully recruited into the CT-26 tumor tissues by the ICD-based tumor cell-specific immune responses (Figure 4C). Finally, the tumor growth of the PBS-, PLGA_7K_ NPs, DOX-HCl-, DOX-PLGA_7K_ NP-, or DOX-PLGA_12K_ NP-treated CT-26 tumor-bearing mice was measured for 28 days after single-direct tumor injection (Figure 4D). As a control, the PBS- or PLGA_7K_ NP-treated mice were all dead within 22 days, wherein mice with a tumor size of 2000 mm^3^ were counted as dead (*n* = 5 per group). In contrast, the DOX-HCl-treated group (*n* = 13) effectively suppressed the tumor growth rate up to 28 days, and the average tumor volume was 140 mm^3^. Furthermore, the DOX-PLGA_7K_ NP- or DOX-PLGA_12K_ NP-treated groups (*n* = 13 per group) showed effective tumor growth suppression compared to that of the PBS-treated group up to 28 days (average tumor volume of 477 mm^3^ and 577 mm^3^, respectively). Each group showed no changes in animal survival for 28 days (Supplementary Materials Figure S4A). Next, we analyzed the tumor-suppressed mice (tumor volume < 300 mm^3^) in each group based on the tumor volume on Day 28. The DOX-HCl-, DOX-PLGA_7K_ NP-, and DOX-PLGA_12K_ NP-treated groups showed that 84.6%, 53.8%, and 30.7% of the mice had suppressed tumor growth, respectively (Figure 4E). As a control group, however, the PBS- or PLGA_7K_ NP-treated groups showed no tumor-suppressed mice. Based on these results, it is obvious that the tumor growth of the CT-26 tumors can be effectively inhibited based on the DOX-induced cytotoxic effect of DOX-PLGA_7K_ NPs and DOX-PLGA_12K_ NPs. However, adaptive immunity would depend on the release kinetics of DOX from the PLGA NPs, wherein a high concentration of free DOX can induce strong ICD and tumor suppression in the primary tumor but adaptive immunity (e.g., CTLs) would not effectively occur. This is because a high concentration of DOX can induce a suppressive or inhibitory effect on immune cells, such as mature DCs and T-cells [29].

### 3.4. Immune-Memory Effect Evaluation

**Figure 5:**
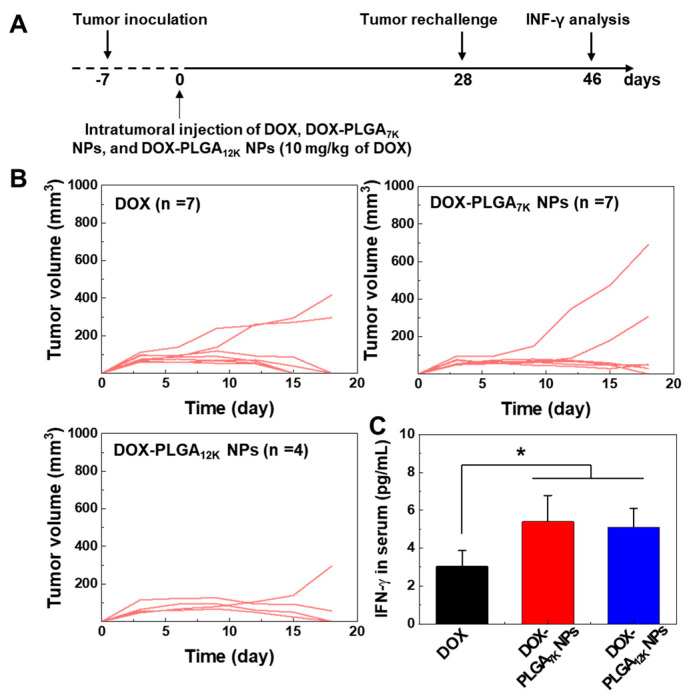
Immune-memory effect evaluation. (**A**) The illustration of an in vivo tumor re-challenging schedule. (**B**) Tumor growth after the CT-26 tumor cell re-challenge in the tumor-suppressed mouse, which were treated with DOX-HCl-, DOX-PLGA_7K_ NPs-, or DOX-PLGA_12K_ NPs. (**C**) Quantified amount of secreted INF-γ in the serum on Day 18 after the CT-26 tumor cell re-challenge. An asterisk (*) indicates a difference at the *p* < 0.05 significance level.

An important feature of cancer immunotherapy is its ability to help the immune system gain a memory effect against a tumor cell-specific antigen, critical for preventing metastasis and recurrence of the tumor. Thus, we evaluated the immune memory effect of tumor-suppressed mice after treating with DOX-HCl-, DOX-PLGA_7K_ NPs, and DOX-PLGA_12K_ NPs. The secondary CT-26 tumors were re-challenged at 28 days after intratumoral injection of DOX-HCl-, DOX-PLGA_7K_ NPs, or DOX-PLGA_12K_ NPs to primary CT26-tumors. The tumor growth of secondary CT-26 tumors was monitored for 18 days, and the INF-γ in the serum of the CT-26 tumor-bearing mice were analyzed (Figure 5A). First, we observed the tumor growth of the secondary CT-26 tumor in tumor-suppressed mice after the first treatment of DOX-HCl-, DOX-PLGA_7K_ NPs, and DOX-PLGA_12K_ NPs (10 mg/kg of DOX). The tumor-suppressed mice treated with DOX-HCl or DOX-PLGA_7K_ NPs showed widespread tumor rejection with 5 out of 7 mice rejecting the tumors. Three out of four mice treated with the DOX-PLGA_12K_ NPs also rejected the tumors (Figure 5B). In survival studies, animals were monitored for 18 days after a tumor re-challenge, wherein it showed no survival changes (Supplementary Materials Figure S4B). We next evaluated the INF-γ in the serum of the CT-26 tumor-bearing mice to understand the antitumor immune memory effect after DOX-HCl-, DOX-PLGA_7K_ NP-, and DOX-PLGA_12K_ NP-based tumor-growth inhibition. Th1 cytokines, including INF-γ, which are representative markers of cellular immunity, serve vital roles in cancer immunotherapy [30,31]. To measure INF-γ, the blood of the CT-26 tumor-bearing mice was collected at 18 days after the re-challenge of the CT-26 tumor cells, followed by separating of serum using centrifugation. Compared to the DOX-HCl-treated mouse, the serum levels of INF-γ were significantly increased when the mouse was treated with DOX-PLGA_7K_ NPs and DOX-PLGA_12K_ NPs, indicating that antitumor immunity was successfully established by the re-challenge of the CT-26 tumor cells (Figure 5C). Furthermore, it was further supported that empty PLGA NPs without DOX could not stimulate the secretion of inflammatory cytokines and immune-suppressive factors, such as IFN- γ, TNF-α, IL-1β, IL-12, IL-2, and IL-6, after treatment [32,33]. Therefore, these results demonstrate that the treatment of the DOX-PLGA_7K_ NPs and DOX-PLGA_12K_ NPs as well as DOX-HCl could successfully establish tumor-specific immunological memory, resulting in eliciting an antitumor effect.

## 4. Conclusions

Conventional cancer vaccines, including peptides or protein antigens, may show diverse efficacies between different types of tumors due to their various antigen expression properties. In situ cancer vaccines that utilize tumor-associated antigens released by the ICD of tumor cells might stimulate antitumor immune responses against multiple types of heterogeneous tumors. Compared with the currently utilized chemotherapeutic agents in cancer immunotherapy, nanomedicines that can provide controlled release of chemotherapeutic agents might provide several advantages for overcoming several issues in cancer immunotherapy. In this study, we hypothesized that the ICD in the tumor tissue could be controlled by using different release kinetics of DOX-loaded PLGA NPs, resulting in eliciting different antitumor immune responses. DOX was successfully encapsulated in both PLGA_7K_ NPs and PLGA_12K_ NPs, resulting in different release kinetics in vitro. Although both the DOX-PLGA_7K_ NPs and DOX-PLGA_12K_ NPs showed an initial burst release of DOX, the DOX released from the DOX-PLGA_7K_ NPs and DOX-PLGA_12K_ NPs was observed up to 10 days and 30 days, respectively. Based on the different DOX release rates from the DOX-PLGA_7K_ NPs and DOX-PLGA_12K_ NPs, the CT-26 tumor cells showed a different cytotoxicity and HMGB1 secretion. Furthermore, the DOX-PLGA_7K_ NPs and DOX-PLGA_12K_ NPs showed sustained release of DOX into the CT-26 tumor tissue after intratumoral injection. Finally, the DOX-PLGA_7K_ NPs and DOX-PLGA_12K_ NPs could inhibit tumor growth and stimulated tumor-specific immune responses, including DC maturation and tumor-infiltration of CTLs, resulting in establishing a tumor-specific immunological memory effect. We expect that the controlled release of ICD-inducible chemotherapeutic agents using different types of PLGA NPs can provide potential in precision cancer immunotherapy by controlled tumor-specific immune responses, thus improving its therapeutic efficacy.

## Supplementary Materials

The following are available online at https://www.mdpi.com/1999-4923/12/12/1165/s1, Figure S1: Magnified time-dependent cellular uptake images of DOX-HCl-, FITC-labeled DOX-PLGA7K NP-, and FITC-labeled DOX-PLGA12K NP-treated CT-26 tumor cells. Blue channel: DAPI, Red channel: DOX, Green channel: FITC-labeled PLGA NPs. The scale bar indicates 5 μm, Figure S2: (**A**) The cell viability of CT-26 tumor cells treated with various concentrations 0.1 to 100 µg/mL of PLGA7K NPs and PLGA12K NPs for 48 h. (**B**) Western blot image and band intensity of HMGB1 which was released in the cell culture medium. CT-26 tumor cells were treated with DOX-HCl, DOX-PLGA7K NPs (5 µM of DOX), or PLGA7K NPs for 48 h, Figure S3: Whole-body DOX fluorescence images of the CT-26 tumor-bearing mice, Figure S4: (**A**) In vivo survival rate of the CT-26 tumor-bearing mice after PBS, DOX, DOX-PLGA7K NP, and DOX-PLGA12K NP treatment. (**B**) In vivo survival rate after a CT-26 tumor cell re-challenge in the tumor-suppressed mice that were treated with DOX-HCl-, DOX-PLGA7K NPs, or DOX-PLGA12K NPs.
