# Supplementary material for: Doxorubicin-Loaded PLGA Nanoparticles for Cancer Therapy: Molecular Weight Effect of PLGA in Doxorubicin Release for Controlling Immunogenic Cell Death
Source: Pharmaceutics. 2020 Nov 29;12(12):1165. doi: 10.3390/pharmaceutics12121165 (PMC7759870; doi:10.3390/pharmaceutics12121165)
Supplement: Supplementary file 1 [file pharmaceutics-12-01165-s001.pdf]

pharmaceutics

Supplementary Materials: Doxorubicin-Loaded PLGA Nanoparticles for Cancer Therapy: Molecular Weight Effect of PLGA in Doxorubicin Release for Controlling Immunogenic Cell Death

Yongwhan Choi, Hong Yeol Yoon, Jeongrae Kim, Suah Yang, Jaewan Lee, Jiwoong Choi, Yujeong Moon, Jinseong Kim, Seungho Lim, Man Kyu Shim, Sangmin Jeon, Ick Chan Kwon and Kwangmeyung Kim

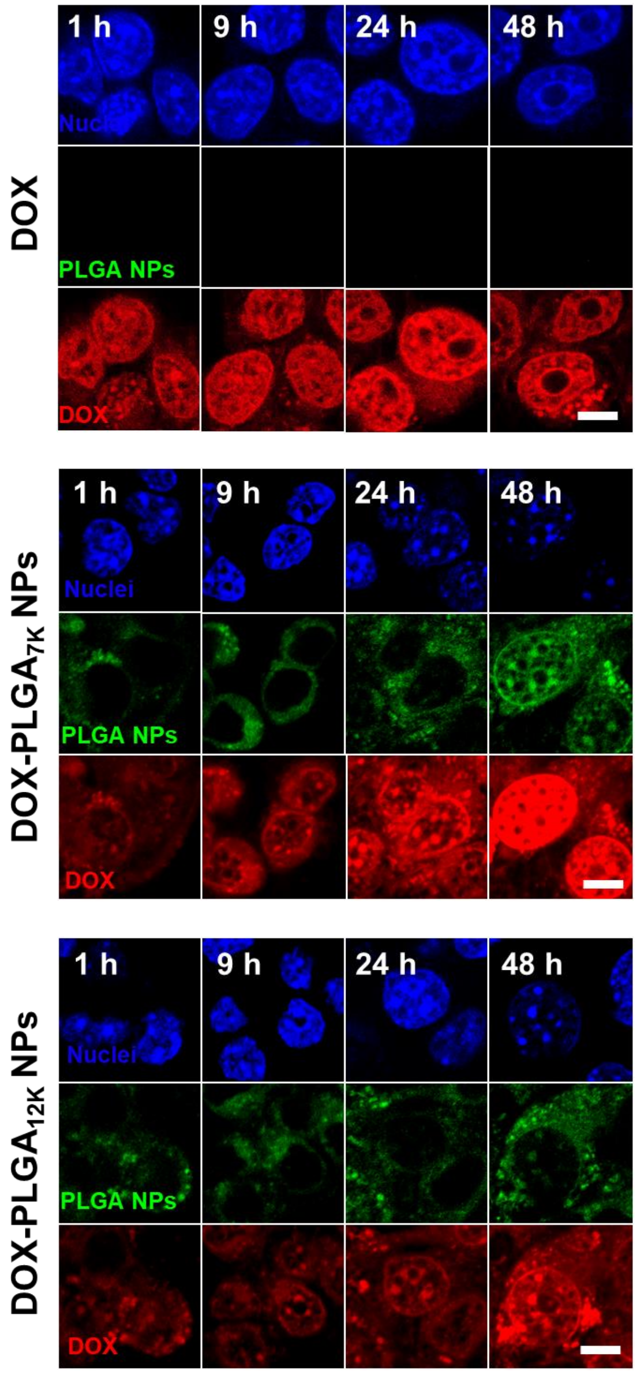

Figure S1. Magnified time-dependent cellular uptake images of DOX-HCl-, FITC-labeled DOX-PLGA7K NPs-, and FITC-labeled DOX-PLGA12K NPs-treated CT-26 tumor cells. Blue channel: DAPI, Red channel: DOX, Green channel: FITC-labeled PLGA NPs. The scale bar indicates 5 μm.

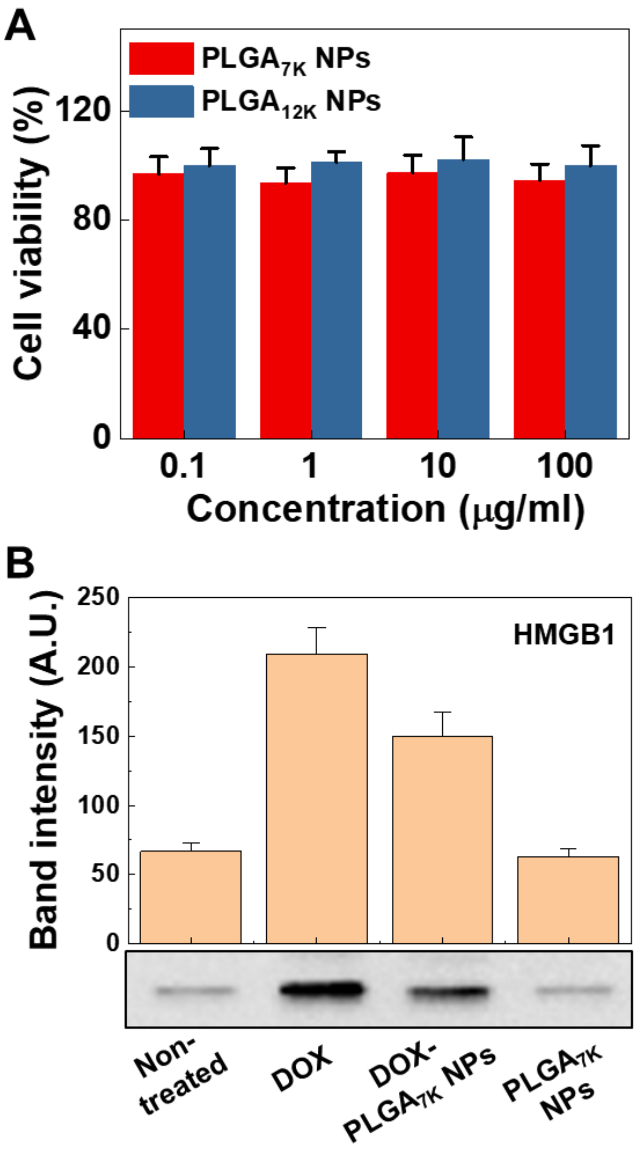

Figure S2. (A) The cell viability of CT-26 tumor cells treated with various concentrations 0.1 to 100 μg/ml of PLGA7K NPs and PLGA12K NPs for 48 h. (B) Western blot image and band intensity of HMGB1 which was released in the cell culture medium. CT-26 tumor cells were treated with DOX-HCl, DOX-PLGA7K NPs (5 μM of DOX), or PLGA7K NPs for 48 h.

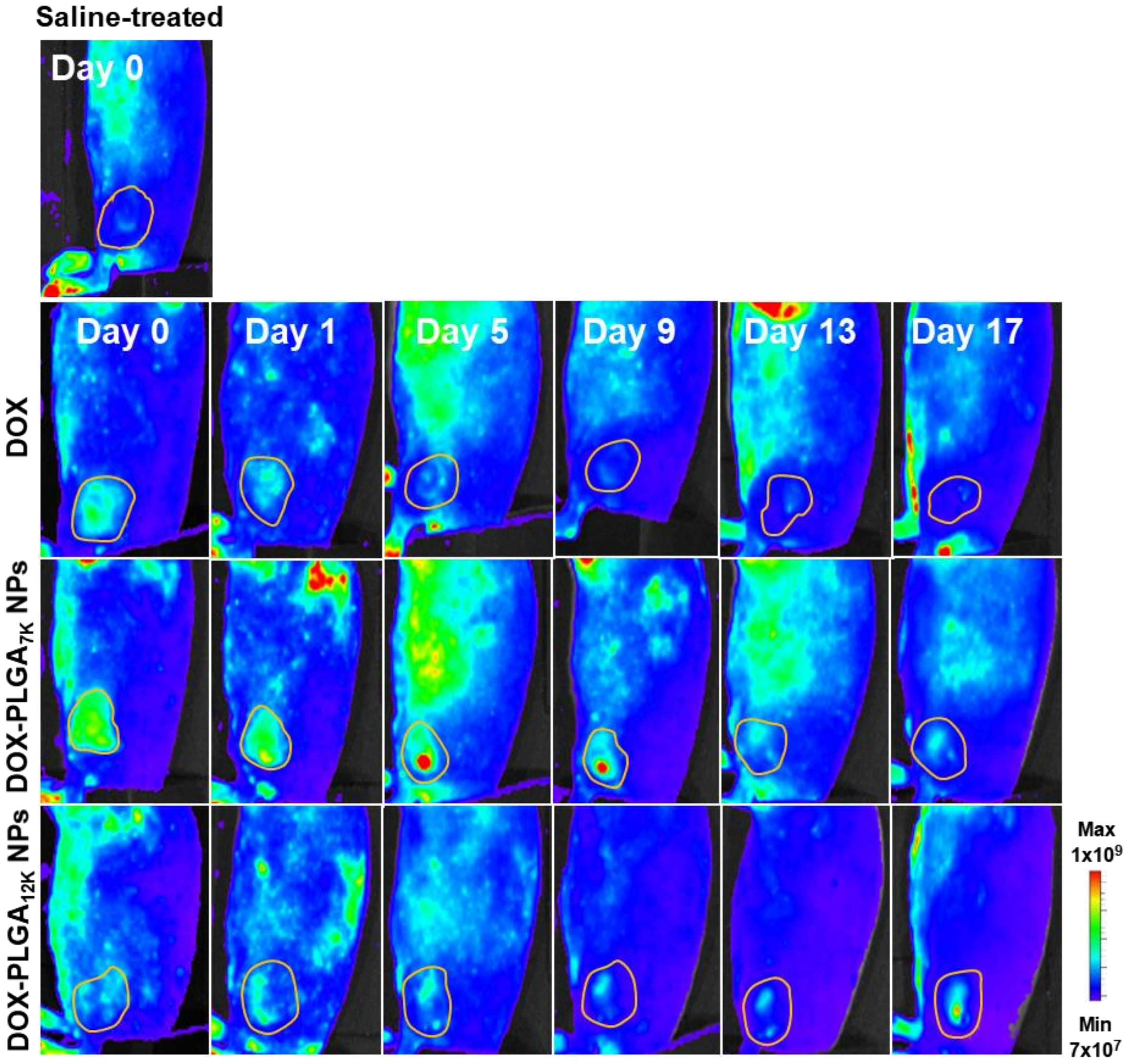

Figure S3. Whole-body DOX fluorescence images of CT-26 tumor-bearing mouse.

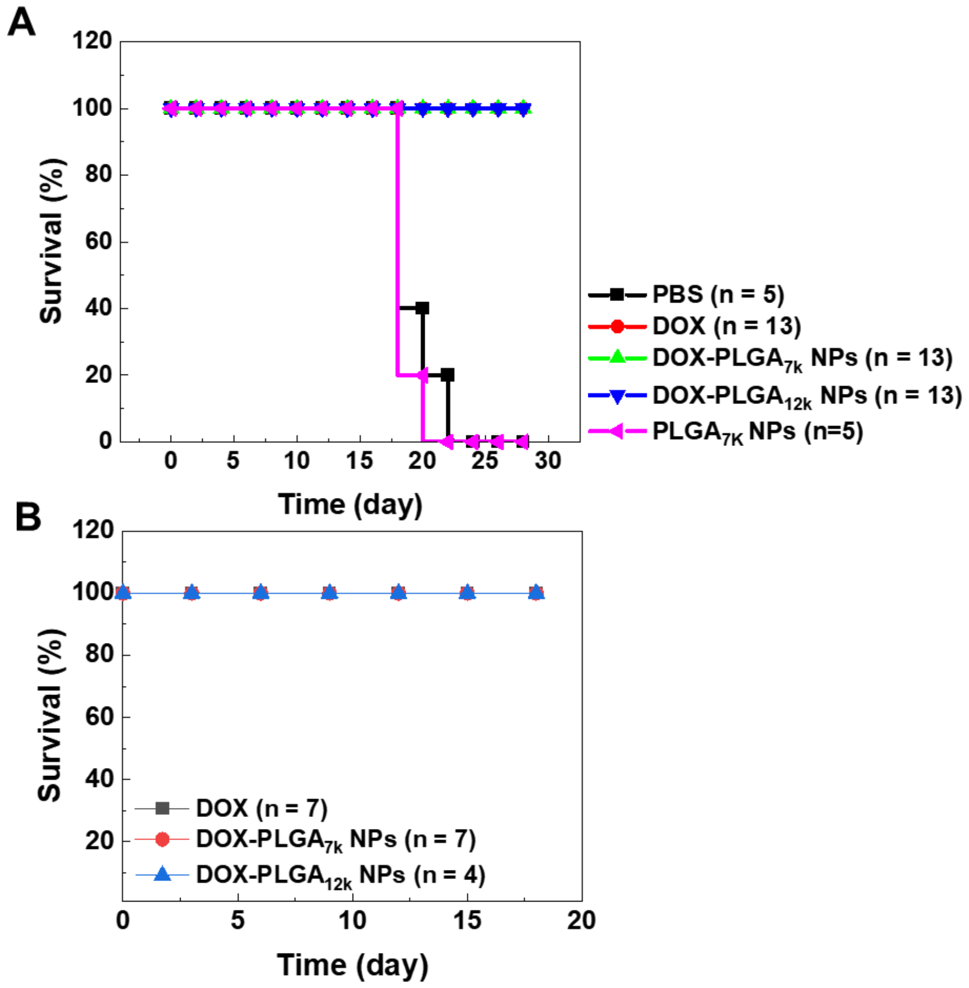

Figure S4. (A) *In vivo* survival rate of CT-26 tumor-bearing mice after PBS, DOX, DOX-PLGA$_{7K}$ NPs, and DOX-PLGA$_{12K}$ NPs treatment. (B) *In vivo* survival rate after CT-26 tumor cell rechallenging to the tumor-suppressed mouse which were treated with DOX-HCl-, DOX-PLGA$_{7K}$ NPs-, or DOX-PLGA$_{12K}$ NPs.
